# Prevalence and associated risk factors of acute respiratory tract infections among under-five children in a tertiary hospital in Nigeria

**DOI:** 10.1186/s12887-025-06193-4

**Published:** 2025-10-29

**Authors:** Fatima Ishaq Abubakar, Oche Mansur Oche, Aminu Umar Kaoje, Bilkisu Ilah Garba, Mikailu Jangebe Abubakar, Hadiza Kubra Ahmed

**Affiliations:** Usmanu Danfodiyo University Teaching Hospital, Sokoto, Nigeria

**Keywords:** Acute respiratory infection, Under-five children, Nigeria, Tertiary hospital, Risk factors, Prevalence, Home environment

## Abstract

**Background:**

Acute respiratory tract infections (ARIs) remain a significant public health concern, contributing to childhood morbidity and mortality, particularly in low- and middle-income countries. This study aimed to determine the prevalence of ARI and identify associated risk factors, including home environmental exposures, among under-five children receiving care at a tertiary hospital in Nigeria.

**Methods:**

A hospital-based cross-sectional study was conducted from August to November 2022 in the Department of Paediatrics, Usmanu Danfodiyo University Teaching Hospital, Sokoto, Nigeria. A total of 251 children aged 2–59 months with ARI, diagnosed using Integrated Management of Childhood Illness (IMCI)/WHO criteria, were enrolled via systematic sampling. Inclusion required informed consent and caregiver reliability in reporting home conditions. Children were excluded if their caregivers could not provide environmental information or if ARI diagnosis was not confirmed. Data were collected through structured questionnaires and analyzed using SPSS v23. Descriptive statistics, chi-square tests, and multivariable logistic regression were applied to identify independent predictors (*p* < 0.05).

**Results:**

The prevalence of ARI was 17.0% (95% CI: 12.8–21.9%). Significant associations were observed between ARI and several environmental factors, including type of cooking fuel, toilet facility, water supply, ventilation, kitchen location, and indoor kerosene lamp use (all *p* < 0.05). Socio-demographic factors such as socioeconomic status, parental education, maternal age, and household size, as well as child-related factors including age, low birth weight, breastfeeding practices, immunization status, daycare attendance, and comorbidities, were also significantly linked with ARI (*p* < 0.05). Independent predictors of ARI included indoor smoking (aOR = 4.34, *p* = 0.023), inbuilt kitchen location (aOR = 4.51, *p* = 0.034), unimproved water supply (aOR = 4.59, *p* = 0.025), poor ventilation (aOR = 3.78, *p* = 0.026), unimproved toilet facilities (aOR = 15.32, *p* = 0.007), and indoor mosquito coil use (aOR = 8.44, *p* = 0.024).

**Conclusion:**

This study highlights a high prevalence of ARI among U5, linked to modifiable household and environmental factors. Families should be encouraged to use clean cooking fuels, improve ventilation, and reduce indoor pollutants like mosquito coils and tobacco smoke. These findings support community education and further research to guide effective, child-focused public health interventions.

## Introduction

Acute respiratory tract infections (ARIs) are responsible for approximately 3.9 million deaths globally, accounting for 16.0% of all deaths, and remain one of the leading causes of morbidity and mortality among children under five (U5) years of age worldwide [1, 2]. In low-income countries (LICs), ARIs contribute to an estimated 1.3 million deaths annually among U5 children [3]. A significant proportion of these deaths—around 50.0%—occur in developing countries such as Nigeria, where ARIs contribute approximately 19.0–20.0% of all child deaths in Africa [4].

Globally, ARIs account for about 3.5% of the total disease burden, with U5 children experiencing 6–8 episodes annually. These infections are responsible for 30–50% of hospital visits, 30–40% of hospital admissions, and contribute to roughly 70% of morbidity among U5 children in developing nations [1, 5]. In Sub-Saharan Africa, ARIs represent around 6% of the total childhood disease burden—higher than that from cancer, diarrheal diseases, malaria, or HIV infections [6].

The impact of ARIs extends beyond clinical outcomes, resulting in multidimensional consequences, including economic losses due to healthcare utilization, caregiver time, family disruptions, prolonged hospital stays, decreased productivity, and increased reliance on empirical treatment. These factors collectively contribute to the global rise in antimicrobial resistance [7]. Additionally, inequitable access to healthcare, particularly among socioeconomically disadvantaged populations such as children under five, increases vulnerability to ARIs and worsens health disparities [8].

According to the World Health Organization (WHO), the incidence rate of ARI in U5 children in developing countries ranges from 15 to 21%, with more than two-thirds of the global burden concentrated in Africa. This places further strain on limited health resources and exacerbates socioeconomic inequalities [9]. A 2015 report by the International Vaccine Access Center ranked Nigeria as the second-largest contributor to the global burden of child pneumonia, a leading cause of under-five mortality [10, 11].

Despite the substantial burden of acute respiratory infections (ARIs) among under-five (U5) children in Nigeria, data on their prevalence and associated risk factors remain limited. In particular, home environmental exposures—such as indoor air pollution from solid fuel combustion, poor ventilation, overcrowding, and inadequate sanitation—play a critical role in the onset and severity of ARIs but are often under-researched in low- and middle-income countries [12–14]. This gap is concerning, given the strong evidence linking poor housing quality to elevated ARI risk. For instance, studies in Nigeria have reported significant associations between risk factors such as indoor environmental conditions and the incidence of ARIs in hospitalized children [15–17]. Understanding these determinants is essential for informing locally appropriate public health interventions. In light of these concerns, this study aimed to determine the prevalence of ARIs and their associations with both household environmental exposures and other risk factors among U5 children accessing care at a tertiary hospital in Sokoto State, Nigeria.

### Aim and objectives

This study aimed to:

- Determine the prevalence of ARIs among children aged 2–59 months accessing care at the Usmanu Danfodiyo University Teaching Hospital, Sokoto, State, Nigeria;
- Identify exposures in the home environment; and
- Assess factors associated with ARIs among under-five children accessing care at a tertiary hospital in Sokoto State, Nigeria

## Materials and methods

### Study population and setting

This cross-sectional study was conducted at the Department of Paediatrics, Usmanu Danfodiyo University Teaching Hospital (UDUTH), Sokoto, Nigeria, over a three-month period (2nd August 2022 to 2nd November 2022).

The study population comprised children aged 2 to 59 months who were diagnosed with acute respiratory tract infection (ARI) at the Paediatric Outpatient Clinic (POPC) and Emergency Paediatric Unit (EPU). These children were evaluated and enrolled during routine consultations or admissions.

## Eligibility criteria

Children were eligible for inclusion if they were aged 2 to 59 months and met the diagnostic criteria for ARI based on the Integrated Management of Childhood Illnesses (IMCI)/WHO case definition for ARI (IMCI/WHO/UNICEF, 2014) [18]. Participants were recruited from the Department of Paediatrics (POPC/EPU), Usmanu Danfodiyo University Teaching Hospital (UDUTH), a tertiary facility, in Sokoto, Nigeria. Children whose primary caregivers (mothers or legal guardians) provided informed consent and were able to reliably report on household and environmental conditions were included. Children were excluded if their caregivers could not provide adequate information about the home environment, or if the children did not meet the IMCI diagnostic criteria for ARI.

### Sample size determination

The minimum sample size was calculated using the formula [19]:

$$\mathrm n=\mathrm z^2\mathrm{pq}/\mathrm d^2$$

### Assumptions

- Confidence level (z): 1.96.
- Estimated prevalence of ARI among under-five children in Nigeria (p): 19.3% (0.193) [20].
- Complement of p (q): 1 − *p* = 0.807.
- Desired precision (d): 0.05.

Substituting the values:

$$n\;=\;\left(1.96\right)^2\;\times\;0.193\;\times\;0.807\;/\;\left(0.05\right)^2\;=\;239$$

Adjusting for a 95% response rate:

$$\mathrm{nf}\;=\;\mathrm n\;/\;0.95\;=\;239\;/\;0.95\;=\;251$$

Thus, 251 participants were recruited using systematic sampling.

### Sampling technique

A systematic sampling technique was employed to recruit participants from both outpatient and emergency paediatric units. The average number of eligible under-five children seen weekly in the POPC was approximately 75, leading to an estimated sampling frame of 900 children over the three-month study period. Using the formula:

$$\mathrm{Sampling}\;\mathrm{interval}=\frac{\mathrm{Total}\;\mathrm{sampling}\;\mathrm{frame}}{\mathrm{Sample}\;\mathrm{size}}=\frac{900\approx4}{251}$$

Participants were selected at every fourth eligible child seen in the clinic. The first participant was randomly selected using a balloting technique from among the first four eligible children. Subsequent participants were enrolled by adding the sampling interval (four) to the serial number of the previously enrolled subject. Similarly, the EPU admitted an average of 7 eligible under-five children daily, giving an estimated frame of 588 children over three months. The calculated sampling interval for the EPU was approximately 2. Accordingly, every second eligible child was selected using the same random-start method.

### Operational definitions

I. Case definition for ARI was based on the Integrated Management of Childhood Illnesses WHO/IMCI 2014 guidelines [18]. These criteria were selected for their global comparability and clinical applicability, even in tertiary care settings. These classified children who presented with cough or difficulty breathing less than 2 weeks as:
  a Upper respiratory tract infection (URTI): Presence of cough, nasal discharge, and/or low-grade fever;
  b Lower respiratory tract infection (LRTI):
    Pneumonia: any child, 2 months to 5 years of age with cough or difficulty breathing and fast breathing greater than 50 breaths/minute and (2-12months; with fast breathing greater than 40 breaths/minute (1–5 years); and
    Severe pneumonia: any child 2 months to 5 years of age with cough or difficulty breathing and any of the following general danger signs; unable to drink or breastfeed, vomits everything, convulsions, lethargic or unconscious or chest in drawing or stridor in a calm child.
II. Socio-economic Status Assessment
  Socio-economic status was determined using the Oyedeji socioeconomic classification system [21]. This method assigns scores based on the educational attainment and occupation of both parents. Each parent’s education and occupation were scored on a scale from 1 to 5, with 1 representing the highest level (e.g., tertiary education and professional occupation) and 5 representing the lowest level (e.g., no formal education and unskilled or unemployed). The average of the four scores (two for each parent) was computed and rounded to the nearest whole number to determine the child’s social class. Social classes were further grouped as follows:
  Class I: Upper socioeconomic status
  Classes II and III: Middle socioeconomic status
  Classes IV and V: Lower socioeconomic status
III. Type of biomass fuel used for cooking, was categorized as follows [22]:
  a clean fuel (electricity, liquefied petroleum gas, natural gas and biogas)’ and
  b unclean/polluted fuel (coal/lignite, charcoal, kerosene, wood, straw/shrub/grass, agricultural crops and animal dung).
IV. Toilet facilities was categorized as follows [23]:
  a Improved sanitation facilities were defined as those that hygienically separate human waste from human contact. This included flush or pour-flush to piped sewer system, septic tank pit latrines, ventilated-improved pit latrines, or pit latrines with slab or composting toilets; and
  b Unimproved sanitation facilities included public or shared-use toilets, pit latrines without slabs or open pits, bucket latrines, hanging latrines or open defecation.
V. Source of water supply: was categorized as [23]:
  a Improved source of drinking water; defined as those that are likely to be protected from outside contamination, and from faecal matter in particular, included (piped into dwelling, piped to yard/plot, public tap/standpipe, piped to neighbor, tube well or borehole, protected well, protected spring, rainwater, tanker truck, cart with small tank)’ and
  b Unimproved source of drinking water included unprotected wells, unprotected springs, surface water (e.g., river, dam or lake), vendor-provided water, bottled water (unless water for other uses is available from an improved source) and tanker truck-provided water
VI. Immunization history which was assessed through mothers’ recall and/or immunization card record as recommended by WHO [24]:
  a Fully immunized: defined as when the child had received one dose of bacilli Calmette-Guerin (BCG), three doses of polio vaccine (excluding oral polio vaccine (OPV) given at birth), three doses of pentavalent and one dose of measles vaccine by 12 months of age.
  b Partially Immunized: defined as child who missed at least any one of the above doses of vaccine
  c Un- immunized or Zero-dose: defined as child who had not received any vaccine by 12 months of age.
    For this study, Immunization status was categorized into Complete and Incomplete Immunization where Complete Immunization included fully immunized children while Incomplete Immunization included partially and un-immunized children.
VII. History of exclusive breastfeeding defined as a child fed only breast milk, except taking the vitamins, mineral supplements, or medicines, until 6 months of age [25].
VIII. History of complementary feeding defined as the process starting other feeds at the age of 6 months when breast milk alone is no longer sufficient to meet infants’ nutritional requirements, resulting in the need for other foods and liquids along with breast milk [26].

## Data collection

Data were collected using:

- A pre-tested interviewer-administered questionnaire, and.
- The Integrated Management of Childhood Illnesses (IMCI)/WHO case definition for ARI (IMCI/WHO/UNICEF, 2014) [18].

The questionnaire, comprising 34 stem questions, was divided into three sections and included both open- and closed-ended questions. It was translated into Hausa, back-translated into English, and revalidated by a language specialist at Usmanu Danfodiyo University, Sokoto.

- Section A: Collected information on socio-demographic and home environment characteristics.
- Section B: Captured physical examination data such as weight (kg), height/length (cm), weight-for-length/height (W/L-H), z-scores, mid-upper arm circumference (MUAC), pedal edema, and temperature (°C).
- Section C: Documented findings from chest examination and confirmed ARI status using the IMCI/WHO definition.

Variables

- Dependent Variable: Presence of ARI (yes/no)

- Independent Variables:
  - Home environment: Overcrowding, cooking fuel type, kitchen location, ventilation, number and placement of windows, parental smoking habits.
  - Child-related factors: Age, sex, immunization status, exclusive breastfeeding, complementary feeding, weaning practices, daycare attendance, and comorbidities (e.g., diarrhea, measles, meningitis).
  - Socio-demographic factors: Maternal age, education, marital status, socioeconomic class, domicile, household size.

## Data collection methods

Mothers or caregivers were interviewed using the standardized questionnaire, followed by clinical and anthropometric assessment of each child. Equipment used included:

- Digital thermometer.
- Electronic weighing scale.
- Locally-fabricated infantometer (for children < 24 months).
- Stadiometer (for children aged 24–59 months).
- Shakir strip (for MUAC).

Each procedure was explained in detail to the caregivers and older children. The clinical examination included general physical and chest assessments.

### Data analysis

Data were cleaned, coded, and analyzed using IBM SPSS version 23.0.

Continuous variables were summarized using median and interquartile range (IQR), while Categorical variables were summarized using frequencies and percentages with 95% confidence intervals (CIs), and Prevalence was calculated using descriptive weighted methods. The Chi-square test was used to assess associations between ARI and various risk factors while logistic regression model was used to determine the factors influencing the occurrence of ARI among U5 children. A p-value ≤ 0.05 was considered statistically significant.

### Ethical considerations

Ethical approval was obtained from the Health Research and Ethics Committee of UDUTH.

Human Ethics and Consent to Participate declarations: All procedures performed in this study was in accordance with Ethical standards of Usmanu anfodiyo University Teaching Hospital (UDUTH) and approval was obtained from the UDUTH Health Research Ethics Committee in accordance with the Declaration of Helsinki (Reference: UDUTH/HREC/2022/1182/V2).

Written informed consent was obtained from each child’s caregiver prior to enrollment. For clarity, the phrase “child-parent pair participants” refers to the child as the study subject, with the parent or guardian providing consent and household information on behalf of the child.

Consent to publish was obtained from all child-parent pair participants included in the study. Participants were informed that the results may be published, and they agreed to the publication of anonymized data.

Clinical trial number: not applicable.

## Results

### Socio-demographic characteristics of under-five children

The majority of the U5 children’s caregivers were aged 21–35 years (78.1%), with a median age of 27 years (IQR: 24–32 years). Nearly all were married (97.6%) and had formal education, with 49.4% attaining tertiary. Their fathers were also largely educated, with 69.6% completing tertiary education. Most households (58.6%) had fewer than five members, resided in urban areas (85.7%), and belonged to the middle socioeconomic class (45.4%) (Table 1).

**Table 1:** Socio-demographic characteristics of under-five children included in the research sites in 2022 (*N* = 251)

Variables	Number (251)	Percentage
Mother’s Age range (years)		
≤ 20	4	1.6
21–35	196	78.1
36–50	51	20.3
Marital status		
Married	245	97.6
Divorced	2	0.8
Widowed	4	1.6
Parity		
1–2	50	19.9
3–4	151	60.2
5–6	42	16.7
≥ 6	8	3.2
Mother/caregiver educational level		
None	0	0.0
Islamic	55	21.9
Primary	4	1.6
Secondary	68	27.1
Tertiary	124	49.4
Father educational level		
None	0	0.0
Islamic	50	20.0
Primary	4	1.6
Secondary	22	8.8
Tertiary	175	69.6
Household size		
< 5	147	58.6
5–9	79	31.4
≥10	25	10.0
Place of residence		
Urban	215	85.7
Rural	36	14.3
Socio-economic status		
Upper	58	23.1
Middle	114	45.4
Lower	79	31.5

Table 2 presents the descriptive and bivariate analysis of demographic, environmental, and clinical factors associated with acute respiratory infections (ARI) among under-five (U5) children (*N* = 251) in 2022. The median age was 22 months (IQR: 12–35 months), with a male-to-female ratio of 1:1.02. The overall ARI prevalence was 17.0% (95% CI: 12.8–21.9%). Upper respiratory tract infections (URTI) predominated among children aged 48–59 months (81.8%), while pneumonia was most prevalent in those aged 24–35 months (32.2%). Severe pneumonia was commonest in children aged 2–11 months (30.4%). Males had a higher proportion of severe pneumonia (22.0%) compared to females (17.7%), whereas females had higher pneumonia prevalence (25.8%). URTI was comparable between sexes.

**Table 2:** Distribution and risk factors of ARI among under-five included in the research sites in 2022 (*N* = 251)

A. Age and Sex Distribution of Acute Respiratory Infections					
Variable	URTI n (%)	Pneumonia n (%)	Severe Pneumonia n (%)		Total (n)
Age (months)					
< 12	32 (46.4%)	16 (23.2%)	21 (30.4%)		69
12–23	42 (63.6%)	10 (15.2%)	14 (21.2%)		66
24–35	25 (44.6%)	18 (32.2%)	13 (23.2%)		56
36–47	10 (62.5%)	4 (25.0%)	2 (12.5%)		16
48–59	36 (81.8%)	8 (18.2%)	0 (0.0%)		44
Sex					
Male	75 (59.1%)	24 (18.9%)	28 (22.0%)		127
Female	70 (56.5%)	32 (25.8%)	22 (17.7%)		124

B. Environmental and Housing Factors					
Variable	Category	URTI n (%)	LRTI n (%)	Test Statistic & *p*-value	
Toilet Facility	Improved	86 (69.9%)	37 (30.1%)	χ² = 14.594, *p* < 0.001	
	Unimproved	59 (46.1%)	69 (53.9%)		
Water Supply	Improved	137 (61.2%)	87 (38.8%)	χ² = 9.820, *p* < 0.001	
	Unimproved	8 (29.6%)	19 (70.4%)		
Cooking Energy	Clean	70 (72.9%)	26 (27.1%)	χ² = 14.621, *p* < 0.001	
	Unclean	75 (48.4%)	80 (51.6%)		
Ventilation	Yes	65 (77.4%)	19 (22.6%)	χ² = 20.062, *p* < 0.001	
	No	80 (47.9%)	87 (52.1%)		
Kitchen Location	Inbuilt	101 (69.7%)	44 (30.3%)	χ² = 31.777, *p* < 0.001	
	Outside	44 (41.5%)	62(58.5%)		

C. Exposure-Related Factors					
Variable	Category	URTI n (%)	LRTI n (%)	Test Statistic & *p*-value	
Indoor Smoking	Yes	35 (66.0%)	18 (34.0%)	χ² = 1.883, *p* = 0.170	
	No	110 (55.6%)	88 (44.4%)		
Mosquito Coil Use	Yes	43 (53.8%)	37 (46.2%)	χ² = 2.856, *p* = 0.240	
	No	102 (59.6%)	69 (40.4%)		
Incense Use	Yes	123 (56.9%)	93 (43.1%)	χ² = 0.432, *p* = 0.511	
	No	22 (62.9%)	13 (37.1%)		
Kerosene Lamp Use	Yes	14 (36.8%)	24 (63.2%)	χ² = 8.038, *p* = 0.005	
	No	131 (61.5%)	82 (38.5%)		
Contact with ARI Case	Yes	99 (61.5%)	62 (38.5%)	χ² = 2.549, *p* = 0.110	
	No	46 (51.1%)	44 (48.9%)		

D. Association between parental sociodemographic factors and ARI among U5					
Variables		URTI n(%)	LRTI n(%)	Test statistical & *P*-value	
Mother’s age					
≤ 20 years		3 (75.0)	1 (25.0)	χ²=11.281, *p* = 0.004	
21–35 years		123 (62.8)	73 (37.2)		
36–50 years		19 (37.3)	32 (62.7)		
Marital status					
Married		140 (57.1)	105 (42.9)	χ²= 1.988, *p* = 0.370	
Divorced		2 (100.0)	0		
Widowed		3 (75.0)	1 (25.0)		
Mother ‘s educational level					
None		0	0		
Islamic		22 (40.0)	33 (60.0)	χ²= 15.725, *p* = 0.001	
Primary		0	4 (100.0)		
Secondary		44 (64.7)	24 (35.3)		
Tertiary		79 (63.7)	45 (36.3)		
Father’s educational level					
None		0	0		
Islamic		14 (28.0)	36 (72.0)	χ²= 25.003, *p* < 0.001	
Primary		4 (100.0)	0		
Secondary		13 (59.1)	9 (40.9)		
Tertiary		114 (65.1)	61 (34.9)		
Household size					
< 6		101 (68.7)	46 (31.3)	χ²= 17.937, *p* < 0.001	
6–9		35 (44.3)	44 (55.7)		
≥ 10		9 (36.0)	16 (64.0)		
Socioeconomic status					
Upper		39 (67.2)	19 (32.8)	χ²=29.334, *p* < 0.001	
Middle		80 (70.2)	34 (29.8)		
Lower		26 (32.9)	53 (67.1)		

E. Association between child’s related factors and ARI among U5					
Variables		URTI n(%)	LTRI n(%)	Test statistical &* P*-value	
Age (months)					
< 12		32 (46.4)	37 (53.6)	χ²= 19.135, p < o.001	
12–23		42 (63.6)	24 (36.4)		
24–35		25 (44.6)	31 (55.4)		
36–47		10 (62.5)	6 (37.5)		
48–59		36 (81.8)	8 (18.2)		
Sex					
Male		75 (59.1)	52 (40.9)	χ²= 0.174, *p* = 0.676	
Female		70 (56.5)	54 (43.5)		
Birthweight					
Unknown		9 (42.9)	12(57.1)	χ² = 8.152, *p* = 0.017	
Low		4 (25.0)	12 (7)		
Normal		132 (61.7)	82 (38.3)		
EBF					
Yes		44(81.5)	10(18.5)	χ² = 15.857, *p* < 0.001	
No		101(51.3)	96(48.7)		
CF *n* = 225					
6 months		103 (65.6)	54 (34.4)	χ² = 20.197, *p* = 0.003	
> 6 months		34 (50.0)	34 (50.0)		
Weaned off BF *n* = 166					
< 2 years		101 (62.3)	61 (37.7)	χ²= 7.014, *p* = 0.003	
≥ 2 years		4 (100.0)	0		
Immunization status					
Complete Immunization		106 (63.5)	61(36.5)	χ²= 6.655, *p* = 0.010	
Incomplete Immunization		39 (46.4)	45 (53.6)		
Hx of contact with ARI					
Yes		99 (61.5)	62 (38.5)	χ²= 2.549, *p* = 0.110	
No		46 (51.1)	44 (48.9)		
Day care Attendance					
Yes		40 (81.6)	9 (18.4)	χ²= 14.212, *p* = 0.000	
No		105 (52.0)	97 (48.0)		
Comorbidities					
None		43 (79.6)	11 (20.4)	χ² = 26.954, *p* = 0.000	
ADDx		61 (54.0)	52 (46.0)		
Measles		4 (33.3)	8 (66.7)		
Meningitis		15 (51.7)	14 (48.3)		
Sepsis		20 (69.0)	9 (31.0)		
Others		2 (14.3)	12 (85.7)		

*URTI  Upper Respiratory Tract Infection, *LRTI * Lower Respiratory Tract Infection, *ARI * Acute Respiratory Infection, χ² Chi-square test statistic, *BF * Breast feeding, *EBF * Exclusive breastfeeding, *Hx * History, *ADDx * Acute diarrheal disease, *CF * Complementary feeding

Environmental characteristics significantly associated with ARI included type of cooking fuel, toilet facility, water source, ventilation, and kitchen location (all *P* < 0.001). Children in households with unimproved toilet facilities or unclean fuel had a higher prevalence of lower respiratory tract infections (LRTI) (53.9% and 51.6%, respectively). Among exposure-related variables, only indoor use of kerosene lamps showed a significant association with ARI (*P* = 0.005), with 63.2% of exposed children presenting with LRTI versus 38.5% of unexposed.

Socioeconomic and parental factors were also significantly associated with ARI: low socioeconomic status (*P* < 0.001), low parental education (*P* = 0.001), younger maternal age (*P* = 0.004), and larger household size (*P* < 0.001). Among child-related variables, ARI showed significant associations with age (*P* = 0.001), birth weight (*P* = 0.017), exclusive breastfeeding (*P* < 0.001), complementary feeding (*P* = 0.003), weaning status (*P* = 0.030), immunization status (*P* = 0.010), day care attendance (*P* < 0.001), and presence of comorbidities (*P* < 0.001). Nearly half (45.0%) of the children had a history of diarrhoeal disease.

Table 3. Presents factors independently associated with ARI among (U5) children.

**Table 3:** Factors associated with occurrence of ARI amongst U5 children included in the research sites in 2022 (*N* = 251)

Variables	Comparison (vs. reference*)	aOR		95% CI	*P*-value
A. Socio-demographic factors and occurrence of ARI amongst the U5 children					
Place of Residence/Domicile	Rural vs. Urban*	0.516		0.174–1.524	0.231
Socioeconomic Class	Middle vs. High*	1.666		0.619–4.481	0.312
	Low vs. High*	6.525		2.787–15.276	0.000
Mother’s Educational Level	Primary vs. No formal*	1.393		0.423–4.596	0.586
	Secondary vs. No formal*	0.602		0.380–0.954	0.031
	Tertiary vs. No formal*	1.798		0.551–5.863	0.331
Father’s Educational Level	Primary vs. No formal*	0.288		0.041–2.029	0.212
	Secondary vs. No formal*	0.948		0.156–5.771	0.954
	Tertiary vs. No formal*	1.375		0.427–4.594	0.586
Mother’s Age	≤ 20 years vs. 21–35 years*	3.752		0.845–9.230	0.995
	≤ 20 years vs. 36–50 years*	5.483		2.236–13.445	0.000
Marital Status	Married vs. Unmarried*	1.434		0.674–3.049	0.349
Household Size	< 6 vs. > 10*	0.029		0.002–0.424	0.000
	6–9 vs. > 10*	1.099		0.298–4.047	0.887
B. Environmental Household Factors Associated with Occurrence of ARI Amongst U5 Children					
Indoor Smoking Habit	Yes vs. No*	4.343		1.229–15.348	0.023
Cross Ventilation	No vs. Yes*	3.783		1.107–12.997	0.034
Location of Kitchen	Inbuilt vs. Outside*	4.512		1.210–16.824	0.025
Type of Cooking Energy	Clean vs. Unclean*	0.189		0.020–1.806	0.189
Type of Water Supply	Unimproved vs. Improved*	4.589		1.198–17.568	0.026
Type of Toilet Facility	Unimproved vs. Improved*	15.322		2.099–111.858	0.007
Indoor Mosquito Coil Use	Yes vs. No*	8.444		1.326–53.778	0.024
Indoor Incense Use	Yes vs. No*	2.766		0.676–11.322	0.157
Kerosene Lamp Usage	Yes vs. No*	2.706		0.579–12.651	0.206
Overcrowding	Yes vs. No*	1.977		0.422–9.263	0.387
C. Child’s related Factors Associated with Occurrence of ARI amongst the U5 children					
Gender	Male vs. Female*	0.691	0.340–1.407		0.309
Age	2–24 vs. 25–59*	2.551	1.134–5.421		0.000
Birth weight	Normal vs. Low*	0.098	0.020–0.488		0.005
Exclusive breastfeeding	Yes vs. No*	0.256	0.096–0.684		0.007
Immunization status	Fully vs. Not fully*	1.555	0.615–3.934		0.351
Day care Attendance	Yes, vs. No*	0.452	0.133–0.715		0.205
Complementary feeding	6months vs. ≥ 6months*	3.239	0.386–4.308		0.279
Weaned off BF	< 2 years vs. 2 years*	0.480	0.257–5.770		0.804
Hx of contact with ARI	Yes vs. No*	3.295	1.041–10.46		0.043
Presence of Comorbidities	Yes vs. No*	10.517	2.167–51.124		0.004

*aOR* Adjusted Odds Ratio, *CI *Confidence Interval, *BF * Breast feeding, *EBF * Exclusive breastfeeding, *Hx * History, *ADDx * Acute diarrheal disease

Section A, highlights socio-demographic predictors. Children from low socioeconomic households had over sixfold increased odds of ARI compared to those from high-income (aOR = 6.525, *P* < 0.001). Maternal education at the secondary level was protective, reducing ARI odds by 40% (aOR = 0.602, *P* = 0.031). Children of mothers aged < 20 years were 5.5 times more likely to develop ARI than those of mothers aged 36–50 (aOR = 5.483, *P* < 0.001). Smaller household size (< 6 members) was associated with 97% reduction in ARI odds (aOR = 0.029, *P* < 0.001).

Section B outlines environmental risk factors. Exposure to indoor cigarette smoke increased the risk of ARI by fourfold (aOR = 4.086, *P* = 0.023). Use of inbuilt kitchens was associated with 4.2-fold increase (aOR = 4.233, *P* = 0.025). Households with unimproved water sources had 4.2 times higher odds of ARI (aOR = 4.221, *P* = 0.026). Absence of cross ventilation tripled the risk (aOR = 3.783, *P* = 0.034). Children living in households with the unimproved toilet were 15 times more likely to develop ARI (aOR = 15.322, *P* = 0.007). Indoor use of mosquito coils increased ARI odds by more than eightfold (aOR = 8.444, *P* = 0.024).

Section C describes child-related predictors. Children aged 2–24 months had 2.6 times greater odds of ARI than those aged 25–59 months (aOR = 2.551, *P* < 0.001), history of contact with ARI cases were over three times more likely to develop ARI (aOR = 3.295, *P* = 0.043). Comorbidities increased ARI odds more than tenfold (aOR = 10.517, *P* = 0.004). Conversely, normal birth weight reduced risk by 90% (aOR = 0.098, *P* = 0.005), and exclusive breastfeeding reduced ARI odds by 74% (aOR = 0.256, *P* = 0.007).

## Discussion

This study reported a 16.8% overall prevalence rate of ARI among under-five children, which is comparable to findings from studies conducted in Port Harcourt, Southern Nigeria (19.3%), Ethiopia (17.7%), and India (14.0%) [20, 27, 28]. These rates align with the global burden of ARI in developing countries, including Nigeria [9]. In contrast, a community-based study in Cameroon reported a significantly higher prevalence of 54.7% among under-five children [29]. This comparatively higher rate may be attributed to the inclusion of a broader and more diverse population, encompassing both rural and urban areas. On the other hand, the 2018 National Nutrition and Health Survey (NNHS) reported a considerably lower prevalence of 4.6% among Nigerian children, with an even lower rate of 1.3% in the North-West region [30]. However, these estimates were based on maternal perception of symptoms rather than clinical diagnosis, which may have contributed to underreporting. It is also noteworthy that this study was conducted between August 2nd and November 2nd, a period that spans the rainy season and the onset of the Harmattan season in Sokoto, Northwestern Nigeria. Although this study did not include seasonal variation as a covariate in the regression analysis due to the data collection period (predominantly rainy season), which limits the assessment of association between seasonality and ARI, existing evidence strongly supports an association between rainy season conditions and increased ARI prevalence [31]. Environmental and climatic factors such as elevated humidity and moist environments which facilitate mold growth and dust mite proliferation, indoor crowding, stagnant water, and increased circulation of respiratory pathogens during the rainy season are well-documented contributors to respiratory infections [31]. A two-year follow-up study in Port Harcourt, Nigeria, found a significantly higher prevalence rate of ARI during the wet (rainy) season [20]. This trend is consistent with findings from Oloyede and Ijezie, which showed that pneumonia admissions among children under five in Uyo, Southern Nigeria, peaked in the rainy months, emphasizing the impact of seasonal weather on respiratory infection rates [32]. Additionally, a recent large-scale evidence by Uttajug et al. supports this association: using Demographic and Health Survey (DHS) data across Sub-Saharan Africa, they found that in Nigeria, extreme rainfall was associated with increased odds of ARI among U5 children [33]. However, we acknowledge that other studies in West Africa have reported peak ARI prevalence during the dry harmattan season, particularly due to exposure to cold dust-laden winds [14, 29, 32]. These differing patterns may reflect regional climate variations and environmental exposures factors. The subtypes of ARI observed showed that the majority had upper respiratory tract infections (URTI) without pneumonia (57.8%), while 22.0% had pneumonia and 20.0% had severe pneumonia. This distribution is similar to findings from Cameroon and India [29, 34]. Children aged 2–23 months had the highest prevalence of ARI (54.0%), particularly those aged 2–11 months who showed the highest prevalence of severe pneumonia (8.4%). This pattern is supported by previous findings indicating increased vulnerability of infants due to immature immune systems and underdeveloped airways [31, 35, 36]. Males had a slightly higher ARI prevalence than females, consistent with other studies, and possibly attributed to immunologic differences, such as the presence of a single X-chromosome in males, which confers less immune protection [31, 37, 38].

This study identified several home environmental exposures significantly associated with acute respiratory infections (ARI) among children under five years of age, including type of cooking fuel, toilet facility, water supply, inadequate ventilation, kitchen location, and kerosene lamp use. Inadequate ventilation was strongly associated with ARI in this study. Similar findings have been reported in Kenya, where residence in houses lacking adequate windows was a significant risk factor for childhood ARI [39]. Studies from Ethiopia and Kashmir have also demonstrated a clear relationship between poor ventilation and increased ARI risk among children [37, 40]. Although Ghimire et al. found no significant association, the biological plausibility remains strong; inadequate ventilation leads to the accumulation of indoor smoke and toxic substances, which damage the immature respiratory epithelium and impair mucociliary clearance, thereby increasing susceptibility to infections [31, 34, 41].

The location of the kitchen also emerged as a significant risk factor. Consistent with Ramani et al.’s findings, having an inbuilt kitchen (versus an outside kitchen) was associated with higher ARI prevalence. Inadequate housing conditions characterized by absence of a separate kitchen and poor smoke outlets result in indoor accumulation of harmful pollutants. Since young children spend extended periods indoors and near their mothers during cooking, they are disproportionately exposed to these toxic substances. This exposure compromises the respiratory tract’s local defenses, increasing ARI risk [31, 42, 43]. Use of kerosene lamps or lanterns was another significant risk factor for ARI, corroborating earlier reports from Ibadan, Nigeria [16]. Kerosene combustion produces indoor air pollutants that contribute to respiratory morbidity, particularly among vulnerable children.

Consistent with prior research, the use of unclean cooking fuels (such as wood, charcoal, kerosene) was strongly associated with ARI [17, 42, 44]. The high concentrations of indoor pollutants generated by these fuels, combined with the prolonged proximity of children to their mothers during cooking, impair ciliary function in the immature lungs and facilitate secondary infections. Notably, Akinyemi et al. found no significant association, likely due to the homogeneity of fuel type usage in their study population, which limited variation. Nonetheless, exposure to unclean fuels clearly raises the vulnerability of young children to ARI, contributing to increased morbidity and mortality [14].

Water supply quality also played a significant role, with unimproved water sources being associated with ARI in our study, consistent with findings from Rwanda and Bangladesh [45, 46]. Improved water quality reduces pathogen transmission via droplets and fomites, interrupting the spread of respiratory infections. Poor sanitation was significantly linked to ARI symptoms, echoing observations from the Philippines [47]. Children living in households with unimproved toilet facilities had a markedly higher prevalence of lower respiratory tract infections (LRTI) compared to those with improved sanitation, consistent with prior research linking poor sanitation to increased infectious disease transmission via fecal contamination and aerosolized pathogens, further exposing under-five children who possess immature immune systems to infections [6, 47].

Indoor smoking habits emerged as a significant risk factor for acute respiratory infections (ARI) in children under five. This finding aligns with extensive prior research demonstrating that secondhand smoke adversely affects children’s respiratory health by damaging the airway epithelium, reducing mucociliary clearance, and impairing local immune defenses [31, 36]. Exposure to tobacco smoke not only increases susceptibility to initial infections but also exacerbates the severity and duration of respiratory illnesses, contributing substantially to childhood morbidity and mortality in low-resource settings. Indoor mosquito coil use was also significantly associated with higher ARI prevalence. The combustion of mosquito coils releases particulate matter, volatile organic compounds, and other chemical irritants that can inflame the respiratory tract mucosa and disrupt normal pulmonary function [12, 38, 44]. This pro-inflammatory environment compromises the respiratory epithelium’s ability to serve as an effective barrier, facilitating pathogen invasion and increasing vulnerability to respiratory infections. Recent studies have corroborated these findings, demonstrating that exposure to mosquito coil smoke significantly increases the risk of respiratory symptoms and infections, particularly in poorly ventilated households [38, 44, 48, 49]. Given the widespread use of mosquito coils as a common vector control method in many endemic regions, these findings raise important public health concerns and highlight the need for safer alternatives to reduce indoor air pollution. Kerosene lamp usage showed a positive correlation with lower respiratory tract infections (LRTI) prevalence; however, this association was not statistically significant after adjusting for other factors. Despite this, kerosene combustion remains a known source of indoor air pollutants such as fine particulate matter, carbon monoxide, and sulfur dioxide, all of which have documented deleterious effects on respiratory health, particularly in young children with developing lungs [16]. The lack of statistical significance in this study may reflect sample size limitations in terms of the relatively smaller number of children exposed to kerosene lamp or confounding from other indoor pollution sources but does not negate the potential respiratory risks posed by kerosene lamp emissions.

Maternal age emerged as a significant determinant of ARI risk, with children born to younger mothers (≤ 20 years) showing a higher likelihood of developing lower respiratory tract infections (LRTI). This may be attributed to younger mothers’ limited experience and potentially reduced knowledge regarding optimal childcare practices, including hygiene and early recognition of illness symptoms. This observation aligns with previous studies reporting that younger maternal age correlates with poorer child health outcomes due to less mature parenting skills and lower health literacy [27, 50]. Parental education, especially maternal education, had a protective effect against ARI. Children whose mothers and fathers attained at least secondary education had a lower incidence of ARI. This protective effect likely reflects better health literacy, improved understanding of hygiene, nutrition, immunization schedules, and a greater likelihood of seeking timely medical care [12, 31, 50–52]. Paternal education was also a significant factor, likely due to its influence on household health decision-making and resource allocation, which indirectly improve children’s living conditions and health outcomes [12, 51].

Larger households were associated with higher rates of ARI. Overcrowding facilitates the spread of respiratory pathogens by increasing close contact between individuals, especially in confined living spaces. Additionally, large households may dilute per capita resources, leading to insufficient nutrition and limited access to healthcare for each child [12, 37, 42, 44]. This finding stresses the importance of addressing living conditions as part of ARI prevention strategies. Socioeconomic status was one of the most critical determinants of ARI occurrence in this study. Children from low-SES households are at greater odds of developing ARI compared to their counterparts from high-SES families. This disparity reflects how poverty intersects with numerous risk factors, including substandard housing, overcrowding, inadequate nutrition, and limited access to quality healthcare services [6, 50, 53]. Lower SES constrains families’ ability to implement preventive measures such as proper sanitation, safe drinking water, and timely immunization, thereby exacerbating vulnerability to infections.

Age was significantly associated with ARI risk, with the highest vulnerability observed in infants under 12 months and children aged 24–35 months. Infants’ immature immune systems render them particularly susceptible to infections, while children in the 24–35 months age group may have increased exposure to infectious agents as they become more mobile and socially interactive [6]. Low birth weight was another significant risk factor, consistent with literature showing that compromised prenatal development impairs lung function and immune responses, increasing infection susceptibility [31, 54]. Exclusive breastfeeding (EBF) was found to be protective against ARI. Children who were exclusively breastfed had significantly lower rates of LRTI compared to those who were not, underscoring the well-established immunological and nutritional benefits of breast milk in enhancing infant immunity and resistance to infections [12, 31, 43, 55]. Appropriate complementary feeding practices, particularly introducing complementary foods at six months, and breastfeeding duration also influenced ARI risk, emphasizing the importance of optimal feeding in supporting child health. Incomplete immunization status was associated with increased ARI occurrence. This finding reinforces the crucial role of vaccination in preventing respiratory infections caused by pathogens such as *Streptococcus pneumoniae*, *Haemophilus influenzae* type b, and *Bordetella pertussis* [56–58]. Additionally, attendance at daycare centers increased the risk of upper respiratory tract infections (URTI), likely due to close interpersonal contact and exposure to contagious pathogens in communal environments [12]. A significant association was observed between ARI and contact with a family member suffering from an acute respiratory illness, consistent with findings from other studies [37, 59]. Close physical proximity between young children and infected family members likely increases the risk of transmission of respiratory pathogens [37]. This pattern is also supported by findings from Ganau et al. and Ujunwa et al., highlighting the role of household-level exposure in the transmission of ARIs among under-five children [12, 56].

Children with comorbid conditions, especially diarrheal diseases, showed significantly higher rates of ARI. This relationship may be due to the compounded effects of malnutrition and immune suppression resulting from concurrent illnesses, which diminish the child’s ability to fight infections [31, 36]. Other illnesses such as measles and meningitis also correlated with elevated ARI risk, highlighting the importance of integrated management of childhood illnesses to improve overall health outcomes.

### Limitations of the study

First, the data collection spanned only three months (from August to November), covering the rainy to early Harmattan season in Northern Nigeria. Since acute respiratory infections, particularly pneumonia, demonstrate seasonal variation—this limited timeframe may have introduced seasonal bias, affecting the generalizability of the prevalence estimate across the year.

Second, although the WHO’s Integrated Management of Childhood Illnesses (IMCI) protocol was used to clinically diagnose ARI, this tool is primarily designed for use at the primary healthcare level. Its application in a tertiary healthcare facility, where more definitive diagnostic tools (e.g., chest radiography) are available, may limit diagnostic precision. Therefore, the possibility of misclassification or under diagnosis cannot be entirely excluded.

Despite these limitations, the study offers important insights into the burden and risk factors of ARI among under-five children in a resource-limited tertiary setting and provides a useful basis for public health interventions and further longitudinal research.

## Conclusion

This study demonstrates a high prevalence of acute respiratory infections (ARI) among under-five children, with significant associations found between ARI and modifiable risk factors, including indoor air pollution, poor ventilation, use of unclean fuels, and certain socio-demographic and child-related variables. These findings highlight the need for coordinated, multi-sectoral strategies that address the underlying social and environmental determinants of child health. Practical implications include strengthening community-based health education to raise awareness of ARI risk factors, particularly around indoor air quality and household practices. Public health campaigns should promote the use of clean cooking energy, discourage indoor smoking and mosquito coil use, and support improved housing standards. At the policy level, these results can inform targeted interventions within maternal and child health programs, and support integration of environmental health considerations into child welfare policies. Ultimately, implementing such strategies could reduce ARI-related morbidity and mortality and advance progress toward national and global child health targets.

## Data Availability

No datasets were generated or analysed during the current study.
